# Nonvitamin, Nonmineral Dietary Supplement Use among Adults with Fibromyalgia: United States, 2007–2012

**DOI:** 10.1155/2017/6751856

**Published:** 2017-07-25

**Authors:** Termeh Feinberg, Christa Lilly, Kim Innes

**Affiliations:** ^1^Center for Integrative Medicine, University of Maryland School of Medicine, Department of Family and Community Medicine, 520 W. Lombard St., East Hall, Baltimore, MD 21201-1603, USA; ^2^West Virginia University School of Public Health, Department of Epidemiology, P.O. Box 9190, Morgantown, WV 26506-9190, USA; ^3^West Virginia University School of Public Health, Department of Biostatistics, P.O. Box 9190, Morgantown, WV 26506-9190, USA; ^4^Center for the Study of Complementary and Alternative Therapies, University of Virginia Health System, P.O. Box 800782, McLeod Hall, Charlottesville, VA 22908-0782, USA

## Abstract

**Background:**

Fibromyalgia (FMS) is a pain condition affecting 2–6% of US adults; effective treatment remains limited. Determinants of nonvitamin, nonmineral dietary supplement (NVNM) use among adults with FMS are not well-studied. We investigated the relation of NVNM use to FMS, and trends, in two nationally representative samples of US adults ≥18 years.

**Methods:**

Data were drawn from 2007 and 2012 National Health Interview Surveys (*N*'s = 20127 and 30672, resp.). Logistic regression was used to examine associations of FMS to NVNM use (past 12 months) and evaluate potential modifying influences of gender and comorbidities. Multivariate models adjusted for sampling design, demographic, lifestyle, and health-related factors.

**Results:**

FMS was significantly higher in 2012 than in 2007 (1.7% versus 1.3%), whereas NVNM use decreased (57% versus 41%; *p* < 0.0001). Adults reporting diagnosis were more likely to use NVNMs within 12 months, 30 days, or ever relative to adults without; positive associations remained significant after controlling for demographics, lifestyle characteristics, medical history, and other confounders (ranges: 2007 and 2012 AORs = 2.3–2.7; 1.5–1.6, resp.; *p*'s < 0.0001).

**Conclusion:**

In this cross-sectional study of two national samples, NVNM use was strongly and positively associated with FMS, highlighting the need for further study.

## 1. Introduction

Fibromyalgia syndrome (FMS) is a rheumatologic neuropathic chronic pain syndrome affecting approximately 2.7% of populations of all ages across the world [1], including an estimated 1.8–6.4% of those in the US [2, 3]. FMS is characterized by a constellation of somatic symptoms (e.g., fatigue, sleep, mood disturbances, and cognitive impairment) that are typically present in addition to widespread pain [4]; the etiology of FMS is largely unknown [4]. Additionally, those with FMS may also experience high rates of comorbidity, such as osteoarthritis [5], autoimmune [6], kidney [7], respiratory [7], and cardiovascular [7, 8] disease, gastrointestinal conditions [7, 9], and headache [1, 7, 10]. Although FMS affects both sexes and people of all ages, the majority of US cases occur in Caucasian, middle-aged women [11, 12].

The chronic widespread pain characterizing FMS is difficult to manage, and many physicians remain unfamiliar with FMS diagnostic criteria and treatment [13] often making it difficult for patients to obtain an accurate FMS diagnosis and appropriate care. Although a lack of efficacy evidence for opioids as FMS treatment exists [14, 15] and opioid overdose deaths have more than tripled since 1999 [16, 17], FMS patients may receive opioid therapy [18]. Common treatments for FMS include antidepressants, anticonvulsants, muscle relaxants, and other pain medications [19]. Nonsteroidal anti-inflammatory drugs, which may carry side effects for as many as 25% of long-term users [20], are not currently recommended for FMS by any guidelines [19]. Although three drugs are FDA-approved for FMS treatment [21], these medications may carry significant side effects for some [22] and are not always effective for all [19]. Thus, patients may turn to complementary health approaches (CHAs, defined as medical/healthcare systems or practices used outside of mainstream medicine [23]) to manage symptoms [24]; back, neck, and joint pain are among the most commonly reported conditions for which patients use CHAs in the US [25, 26].

Nonvitamin, nonmineral dietary supplements (NVNMs) are a type of CHA used by nearly twenty-five percent of US adults with a musculoskeletal disorder [25] and include animal- and plant-derived products (i.e., fish oil, herbal dietary supplements). The extent and efficacy of NVNMs, particularly herbal dietary supplements, are largely unknown [27, 28]. Although a handful of large surveys have documented high rates of herbal and other NVNM use for chronic pain syndromes [24, 25, 29, 30], and anecdotal accounts indicate that a wide range of herbs have been used for the treatment of FMS [31], rigorous investigations regarding the prevalence and correlates of NVNM use in FMS remain limited. To address these gaps, we assessed the relation of FMS to NVNM use in two large, representative samples of US adults and examined potential changes in prevalence rates for NVNM use among those with FMS over a 5-year time period.

## 2. Materials and Methods

### 2.1. Data Sources

Participants for this study were drawn from two nationally representative samples of 23,501 and 34,525 US adults (National Health Interview Surveys (NHIS), 2007 and 2012, resp.). The NHIS is an annual national, cross-sectional household survey of the noninstitutionalized US population and is administered by the Centers for Disease Control and Prevention's National Center for Health Statistics. A supplement (adult alternative medicine) to the core individual, family, and household surveys asked participants about use of a broad range of CHAs, including herbs and other NVNMs, for both years. All questions were administered in a personal interview format. Adult response rates to the NHIS were 78.3% in 2007 and 79.7% in 2012, respectively [32].

The NHIS is the only national, public-use survey that includes comprehensive sets of questions regarding NVNM and other CHA use. Both 2007 and 2012 surveys used a stratified multistage probability design weighted on age, sex, and race/ethnicity, using 2000 and 2010 Census data for each, respectively. Both surveys oversampled Asian, Black, Hispanic, and minority elderly populations. Thus, each person in the covered population had a known nonzero probability of selection. Additional project details have been described elsewhere [32].

### 2.2. Study Population

**Figure 1:**
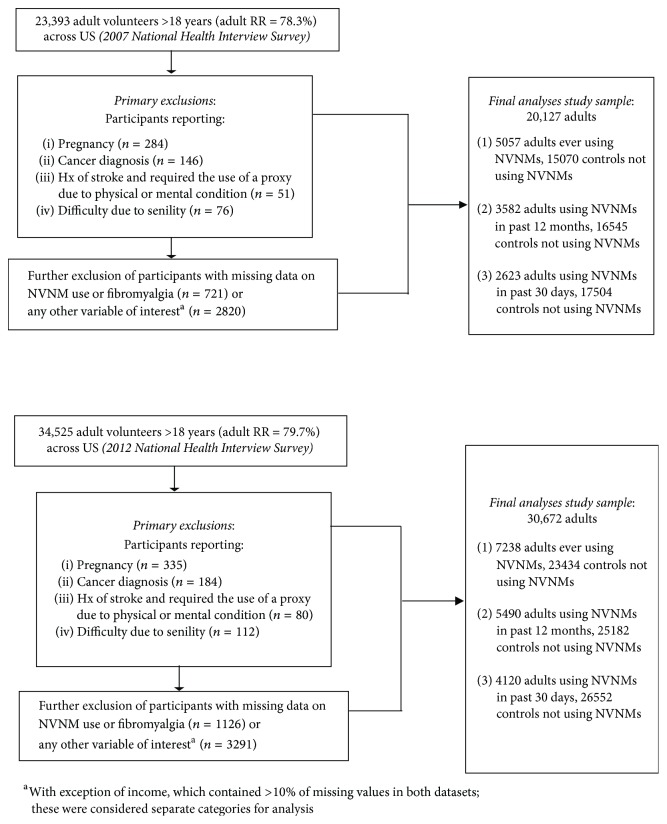
Study flow diagram for nonvitamin, nonmineral dietary supplement (NVNM) use in two national datasets.

Our analysis excluded participants who were <18 years of age, were pregnant, had functional limitation(s) due to senility, had a stroke and used a proxy to complete the interview, or had current cancer; those missing data on key covariates were also excluded, as were participants with extreme values for exercise (>6,000 minutes per week) in order to eliminate potential information bias. Further exclusion of persons with missing data on FMS and NVNM use (at 30 days, at 12 months, or ever using NVNMs) yielded final study samples of 20,127 and 30,672 adults for 2007 and 2012, respectively (Figure 1).

### 2.3. Variables

#### 2.3.1. Outcome Variables

The main outcome variable for this study was the reported use of NVNMs within the past 12 months (Y/N). We additionally assessed use of NVNMs within the previous 30 days (Y/N) and ever (Y/N). Briefly, survey participants were shown a card with a list of NVNMs (for which the majority were comprised of herbs or their compounds; 2007 list: 44 NVNM items with additional queries for up to two combination supplements, and 2012 list: 21 NVNM items with additional queries for up to two combination supplements) and queried for a dichotomous response regarding consumption of any “herbal or other nonvitamin supplements.” NVNMs included those labelled “dietary supplement” and in the form of pills, capsules, tablets, or (2012) liquids (including tinctures) and did not include homeopathic or cannabis products.

#### 2.3.2. Exposure Variable

Our main exposure was fibromyalgia syndrome (FMS) (Y/N), ascertained as an affirmative response to fibromyalgia after being asked “Have you EVER been told by a doctor or other health professional that you had some form of arthritis, rheumatoid arthritis, gout, lupus, or fibromyalgia? Which of these were you told you had?”

#### 2.3.3. Covariates

Demographics, lifestyle factors, health conditions, and medical care-related factors known or suspected to be associated with CHAs, NVNM use, and/or FMS were considered a priori as potential covariates in multivariate models. Demographic factors assessed included age, gender, race/ethnicity, education, employment, income, marital status, geographic region, and place of birth. Additional related factors included insurance status, annual family out-of-pocket medical costs, and delay of care due to concerns over cost. Lifestyle factors included smoking status, alcohol use, exercise, BMI (using the National Institutes of Health clinical classifications scores) [33], health status, and substance abuse. Health conditions included self-reported history of physician-diagnosed diabetes, kidney disease, gastrointestinal disorder, respiratory conditions, dyslipidemia, liver condition, rheumatoid arthritis, cardiovascular disease, hypertension, nonspecific arthritis, gout, migraines, mental health condition, insomnia, and previous cancer diagnosis. These variables are described in greater detail below.

### 2.4. Statistical Analysis

We conducted complete-case analyses using SAS 9.4 (Cary, NC, USA) and used sampling weights to account for complex survey procedures; these were adjusted for the probabilities of selection, nonresponse, and poststratification [34]. We merged NHIS family, person, household, sample adult, and adult alternative health files for each year and measured sample characteristics, including frequencies/prevalence rates of NVNM use (previous or current use, past 12 months, and past 30 days) for 2007 and 2012, respectively; we extrapolated estimates to generate population frequencies using NHIS sampling weights. Weighted *t*-tests and Rao-Scott Chi-square tests were used to determine significant differences by NVNM use status for three outcome time points in both survey years; significant factors were included in models as covariates. We also used a DOMAIN statement to keep our exclusions separate while maintaining the integrity of sampling weights and used Chi-square and *t*-tests to determine significant changes between 2007 and 2012 on weighted frequencies and means of all items, in addition to number of different NVNMs used, physician disclosure of NVNM use, and use of other CHAs. We considered trends between time points significant if there was no overlap in weighted percentage confidence intervals. Differences between participants with versus without missing data were assessed. All *p* values shown are two-sided at *p* ≤ 0.05.

Weighted logistic regressions were used to evaluate the independent associations of FMS diagnosis to NVNM use in the last 30 days or 12 months or ever. All multivariate models were adjusted for demographics and medical care-related factors; additional models also included lifestyle characteristics and health conditions.

Demographic characteristics included age (evaluated as both a continuous and categorical variable (18–24, 25–44, 45–64, 65–74, and 75+ years)), sex (male/female), race (“non-Hispanic White,” “non-Hispanic Black,” “Hispanic,” “Asian,” and “other”), marital status (“married/cohabitating,” “divorced/separated/widowed,” and “single”), education (“≤some HS,” “HS/GED,” “some college/AA/tech,” and “bachelor's degree+”), employment (“employed for pay,” “employed but not for pay,” and “unemployed”), income ($1–$24,999; $25,000–$44,999; $45,000–$74,999; $75,000+; “do not know;” and “missing”), geographic region (“northeast,” “midwest,” “south,” and “west”), and place of birth (US-born/other). Medical care-related factors included insurance status (“uninsured,” “Medicaid,” “Medicare,” “disability,” and “private”), annual out-of-pocket medical costs (“none”; <$500; $500–$1999; $2000–$2999; $3000–$4999; $5000 and over; and “do not know”), and delayed access to care because they “could not afford” or “worried about cost” (yes/no). Additional analyses also controlled for lifestyle-related factors, including BMI (evaluated as continuous and categorical (<18.5 = “underweight,” 18.5–25 = “underweight or normal weight,” 25–29.9 = “overweight,” 30–34.9 = “obese class 1,” and 35+ = “obese classes 2/3”)) [35]; tobacco use (“current smoker,” “former smoker,” and “never smoked”); alcohol use (“none,” “light,” and “moderate to heavy”); substance abuse other than tobacco or alcohol in past year (yes/no); and physical activity (continuous, in minutes/week).

In our fully adjusted models, we also evaluated the potentially confounding influence of self-reported health status (“excellent/very good/good,” “fair,” and “poor”), specific health conditions, and total number of health conditions; this comorbidity index was created from 0–13 participant-reported conditions, including a history of (1) diabetes; (2) kidney disease; (3) gastrointestinal disorder (including history of ulcers, inflammatory/irritable bowel disease, or constipation severe enough to require medication in the past year and/or (2012) abdominal pain, digestive allergy, and/or heartburn in the past year); (4) respiratory conditions (history of asthma or emphysema and/or chronic bronchitis); (5) dyslipidemia; (6) liver condition; (7) rheumatoid arthritis; (8) cardiovascular disease (coronary heart disease, angina, and/or heart attack); (9) hypertension; (10) nonspecific arthritis; (11) gout; (12) migraines; and (13) mental health condition (depression, phobias, and/or being often anxious in past year and/or ever having bipolar disorder). Health conditions were evaluated both collectively and individually; insomnia in the past year and previous cancer diagnosis were evaluated individually.

We also assessed the potential modifying influence of gender and presence of comorbid health conditions (0-1 versus 2+) on the association between FMS and NVNM use at each outcome time point and for each survey year by including the corresponding multiplicative-interaction term in age-adjusted models.

## 3. Results and Discussion

Relative to participants with complete data in both survey years, those with missing data on key covariates were less likely to be employed and report high educational attainment and more likely to be underweight, be older, and indicate poor reported health status (*p*'s ≤ 0.0001). Participant age ranged from 18 to 85 years, averaging 47.5 ± 0.25 and 48.5 ± 0.18 years for 2007 and 2012, respectively. In both years, study participants were predominantly white (72.2% and 69.5%, resp.), female (53.1% and 53.5%), and insured (84.3% and 84.2%); most (56.4% and 57.9%) reported they never smoked cigarettes. Excluding prayer, most participants had used at least one CHA before (69.8% and 68.8%), and a majority had also used natural products other than NVNMs (60.4% and 62.4%) (not shown). In both years, 35% were overweight (mean BMI = 27.4 ± 0.05 and 27.7 ± 0.04, for 2007 and 2012, resp.), and most (63% and 72%) reported at least one chronic health condition, with the number of chronic health conditions increasing from a mean of 1.5 ± 0.02 in 2007 to 1.9 ± 0.01 in 2012.

Table 1 illustrates the distribution of demographic and lifestyle characteristics, stratified by year and NVNM use in the past 12 months; medical-related/health characteristics are displayed in Table 2. The percentage of adults reporting NVNM use (ever) declined significantly between time points (*p* < 0.0001) from 27.1% of the sample population in 2007 (*n* = 5057) to 24.2% in 2012 (*n* = 7238; not shown). In contrast, the percentage of adults reporting use of NVNMs within the last 12 months (19.1% to 18.5%, resp.) and 30 days (13.9% in both years; not shown) remained approximately the same. Prevalence of diagnosed FMS also increased from 1.3% to 1.7% (*p* < 0.0001) (Table 3). Consistent with a slight decrease in overall NVNM use among the general population, overall NVNM use among adults diagnosed with FMS declined from 57% in 2007 to 41% in 2012 (*p* < 0.0001). There were no apparent changes in the number of different combination NVNM supplements taken among those with FMS (mean (SE) for past 30 days = 0.99 (0.20) in 2007, 1.1 (0.38) in 2012, *p* = 0.89).

Between 2007 and 2012, rates of NVNM consumption (past 12 months; Table 1) declined among non-Hispanic White adults (83% to 79%) but increased among those identified as Hispanic (7% to 9%; *p*'s < 0.0001). Overall, the likelihood of NVNM use significantly increased by number of health conditions in both years (Table 2; *p*'s for trend ≤ 0.0001, not shown). In addition, NVNM users (past 12 months) spending under $500 in out-of-pocket medical costs decreased (2007 and 2012: 35.0% and 31.3%, resp.) while NVNM users spending over $3,000 increased (6.2% and 8.3%). This was also noted by an increase in likelihood of NVNM use by out-of-pocket costs for both years (*p*'s for trend = 0.0001; not shown). Lastly, consumption of other natural products (i.e., vitamins/minerals) increased (93% to 96%) among all herbal users between time points.

### 3.1. Relation of FMS to Herbal Use

Nearly 60% of those with FMS reported previous or current NVNM use; 42% indicated using NVNMs in the past 12 months, and 33% in the past 30 days (Table 3). Approximately 3% of all NVNM users had FMS; this rate did not differ between years (Table 4). In 2007, participants with FMS were 3.7 times as likely as those without FMS to use NVNMs for any length of time (OR = 3.7, 95% confidence interval (CI): 2.7, 5.0). After adjustment for demographic, lifestyle, and medical/health factors, those with FMS were 2.7 times as likely to report ever using NVNMs than those without FMS (adjusted odds ratio (AOR) = 2.7, (CI: 1.9, 3.8)). FMS showed a similarly strong positive association to recent NVNM use (12-month and 30-day AORs = 2.3 (CI: 1.5, 3.4) and 2.3 (CI: 1.6, 3.5), resp.). Likewise, FMS was significantly and positively associated with all NVNM use outcomes in 2012, although the magnitude of the associations was lower (2012 AORs: herbal use (ever) = 1.6 (CI: 1.3, 2.0); 12 months = 1.6 (CI: 1.2, 2.0); 30 days = 1.5 (CI: 1.2, 2.0)).

The magnitude of the association of NVNM use to FMS was significantly greater in those with 0-1 comorbid health conditions than in those with 2 or more health conditions (age-adjusted ORs, resp., = 4.7 (CI: 2.2, 9.8) versus 2.8 (CI: 2.0, 3.9; *p* for interaction = 0.008) in 2007; not shown). However, we found no evidence of a modifying effect of multiple comorbidities in 2012 or for gender in either year.

To our knowledge, this is the first large, population-based study to examine the relation of NVNM use to FMS; it is also among the first rigorous studies to investigate the relation of NVNM use to any chronic pain condition in a large sample [25, 29, 30, 36]. Relative to adults without FMS, those with FMS were significantly more likely to report using NVNMs at any time point (within the past 30 days or 12 months or ever) in both 2007 and 2012 survey years; the positive association of FMS to NVNM use remained highly significant even after controlling for a broad array of demographic, lifestyle, and health-related factors.

In our study, the majority of those with FMS indicated using NVNMs at some time point; one-third had used NVNMs in the past 30 days. Previous studies assessing herbal use in FMS reported widely varying rates among FMS patients, ranging from 43% (FMS = 434) to 78% (FMS = 90) [36, 37]. Reported rates among other chronic pain populations have been considerably lower, ranging from 6.8% in a sample of primary care patients using opioids [29] to 15% in a primary care patient population with chronic pain [30]. However, percentages given in prior studies were unweighted and may in part reflect differences in study populations and outcome definitions, rendering comparison with our findings challenging.

Prevalence of diagnosed FMS increased between 2007 and 2012 (1.3% to 1.7%). Despite lower rates than those appearing globally and in some US studies [1–3], this apparent rise in diagnosed FMS may be due in part to increased recognition of FMS by physicians following publication of the 2010 Preliminary ACR updated criteria for fibromyalgia.

In contrast, we found reported NVNM use among adults with FMS declined significantly from 2007 to 2012, paralleling the modest but significant reduction in overall NVNM use observed between these periods. However, the mean number of different combination NVNM supplements used by those with FMS did not differ between time points and averaged lower than that reported in a case-control study of North American women (*N* = 434 with FMS) [36]. In addition, both the observed decline in NVNM use among those with FMS and the positive but more modest association between NVNM use and FMS in 2012 compared to 2007 may also reflect increased availability of FDA-approved FMS medications following the 2007 NHIS survey administration. It also remains possible that both the increase in FMS diagnoses and decrease in odds of NVNM use for FMS over time are also in part the result of promotion for FMS drugs by pharmaceutical companies [38] and increased off-label prescription of other drugs for FMS, potentially including opioids [39, 40].

*Strengths and Limitations*. This investigation was the first study to assess the relation between NVNM use and FMS in a large US population. Strengths of our study include large, nationally representative samples, use of data from two time periods, and the availability of comprehensive information on NVNM use, as well as a broad array of demographic, lifestyle, health-related, and other potentially confounding factors. However, there were some limitations. Perhaps most important, the cross-sectional study design precluded determination of causal relationships. Ascertainment of NVNM use and medical history, including diagnosis of FMS, was reliant on self-report, raising the possibility of recall bias; however, potential underascertainment would likely bias our results toward the null, indicating more conservative estimates.

Although the NVNM use outcome mainly comprised of herbal dietary supplements and was assessed separately from many other nonherbal supplements in both NHIS data collection periods, the nonherbal products coenzyme Q-10, SAM-e, fish oil, and prebiotics/probiotics were included in our main NVNM outcome. Thus, our consideration of a broader NVNM outcome may be more appropriate than a specific herbal supplement use outcome, since reports of individual ingredients were not consistently available between years. There was inadequate capture of certain herbal products within the NVNM outcome. No data were available on consumption of herbal and green teas used for health purposes or on products not labelled (per NHIS requirements for inclusion) “dietary supplement,” including home grown herbs, traditionally prepared herbal products, and bulk herbs/powders sometimes recommended (i.e., by nutritionists, natural foods purveyors) over widely available supplements due to quality and processing concerns. We did not have information on FMS severity or duration, and although the 2012 questionnaire differed from 2007, there were no differences with respect to assessment of fibromyalgia. Based on cognitive testing and input from expert panels, the definitions of certain modalities were modified in 2012 to reduce false-positive responses [34] which may have affected findings. However, these changes were relatively minor and were unlikely to affect our estimates, as these potential false-positives did not vary by disease status.

Given the high prevalence rates of those with FMS using NVNMs, and the strong, positive relationship of FMS to NVNM use which persisted in 2012 despite the availability of approved treatments, additional prospective research is warranted to confirm these findings, to further investigate the prevalence, patterns, and determinants of specific NVNM use in FMS, and to explore the safety and potential efficacy of NVNM products for this still poorly managed condition.

## 4. Conclusions

In the first large cross-sectional study of two nationally representative samples of US adults, reported diagnosis of FMS was strongly and positively associated with NVNM use in both 2007 and 2012 after adjustment for demographic, lifestyle, and health factors. Rigorous prospective studies are needed to confirm these findings and to further explore the patterns, determinants, and potential efficacy of NVNM use in those with FMS.

## Figures and Tables

**Table 1:** Demographic and lifestyle characteristics of 2007 and 2012 national US samples, stratified by NVNM use in the past 12 months, National Health Interview Survey.

Characteristic	2007 (*N* = 20,127)				*p*	2012 (*N* = 30,672)				*p*	Overall *p*^W^
	NVNM Use					NVNM Use					
	Yes	95% CI	Weighted population estimate^*∗*^	No		Yes	95% CI	Weighted population estimate^*∗∗*^	No		
	*N* (%)			*N* (%)		*N* (%)			*N* (%)		
Total	3582 (19.1)	(18.4, 19.8)	16270972	16545 (80.9)		5490 (18.5)	(17.8, 19.2)	16713004	25182 (72.4)		<0.0001
Age in years					<0.0001					<0.0001	<0.0001
18–24	269 (8.2)	(6.8, 9.6)	1332833	1884 (11.7)		391 (7.4)	(6.3, 8.4)	1231081	2637 (11.1)		
25–44	1169 (31.9)	(30.1, 33.7)	5193487	6195 (36.3)		1775 (32.1)	(30.4, 33.7)	5358304	8879 (34.1)		
45–64	1414 (39.8)	(37.9, 41.7)	6476821	5314 (32.3)		2162 (39.1)	(37.5, 40.8)	6542615	8379 (33.1)		
65–75	462 (12.6)	(11.4, 13.7)	2044193	1599 (9.5)		741 (14.0)	(12.8, 15.1)	2332396	2777 (11.4)		
75+	268 (7.5)	(6.5, 8.5)	1223638	1553 (10.2)		421 (7.5)	(6.6, 8.4)	1248608	2510 (10.3)		
*Mean (SE)*	*49.06 (0.35)*	*(48.38, 49.75)*		*47.09 (0.27)*	<0.0001	*49.58 (0.33)*	*(48.93, 50.23)*		*48.24 (0.19)*	<0.0001	0.2794
Sex					0.0001					<0.0001	<0.0001
Male	1535 (43.2)	(41.3, 45.1)	7028291	7644 (47.8)		2341 (42.8)	(41.1, 44.5)	7153927	11565 (47.3)		
Female	2047 (56.8)	(54.9, 58.7)	9242681	8901 (52.2)		3149 (57.2)	(55.5, 58.9)	9559077	13617 (52.7)		
Race/ethnicity					<0.0001					<0.0001	<0.0001
Non-Hispanic White	2716 (82.9)	(81.7, 84.1)	13487880	9419 (69.6)		3979 (78.8)	(77.5, 80.1)	13163299	14637 (67.4)		**∗** **∗** **∗**
Non-Hispanic Black	329 (6.9)	(6.0, 7.8)	1121133	2812 (13.5)		513 (7.6)	(6.7, 8.4)	1266927	4058 (13.3)		
Hispanic	360 (6.7)	(5.9, 7.5)	1091895	3358 (12.8)		622 (9.0)	(8.1, 9.8)	1496127	4796 (14.4)		**∗** **∗** **∗**
Asian	99 (1.8)	(1.4, 2.2)	297318	547 (2.3)		196 (2.6)	(2.2, 3.0)	438850	975 (2.9)		
Other Race/ethnicity	78 (1.7)	(1.3, 2.0)	272746	409 (1.8)		180 (2.1)	(1.7, 2.4)	347801	716 (2.1)		
Marital status					0.088					0.009	<0.0001
Married/ Cohabitating	1903 (52.0)	(50.0, 53.9)	8453944	8507 (51.4)		2865 (52.1)	(50.2, 53.9)	8703339	12354 (49.4)		
Never married	710 (21.0)	(19.2, 22.7)	3411156	3800 (23.1)		1206 (22.8)	(21.2, 24.4)	3813588	6184 (24.7)		
Divorced/sep/ widow	969 (27.1)	(25.5, 28.7)	4405872	4238 (25.6)		1419 (25.1)	(23.7, 26.5)	4196077	6644 (25.9)		
Education					<0.0001					<0.0001	<0.0001
<12th grade	272 (7.0)	(6.1, 7.8)	1131795	3307 (16.5)		417 (6.4)	(5.7, 7.2)	1076502	4420 (15.1)		
HS/GED	808 (22.3)	(20.7, 23.8)	3624376	4830 (29.6)		1043 (18.4)	(17.2, 19.6)	3078409	6860 (26.8)		**∗** **∗** **∗**
Tech training/ some college	1185 (33.4)	(31.6, 35.2)	5436406	4527 (28.5)		1924 (35.3)	(33.6, 36.9)	5896361	7548 (30.7)		
≥Bachelor's degree	1317 (37.4)	(35.4, 39.3)	6078395	3881 (25.4)		2106 (39.9)	(38.1, 41.6)	6661732	6354 (27.4)		
Employment					<0.0001					<0.0001	<0.0001
Employed for pay	2189 (61.6)	(59.8, 63.3)	10016152	9818 (59.5)		3301 (60.2)	(58.5, 61.9)	10065089	13857 (55.4)		
Employed but not for pay	162 (4.8)	(4.0, 5.6)	784425	465 (3.1)		227 (4.3)	(3.7, 5.0)	725979	703 (3.0)		
Unemployed	1231 (33.6)	(31.9, 35.3)	5470395	6262 (37.4)		1962 (35.4)	(33.7, 37.1)	5921936	10622 (41.7)		
Income					<0.0001					<0.0001	<0.0001
$1–$24,999	820 (23.4)	(21.8, 25.1)	3809168	3710 (22.2)		1186 (21.0)	(19.8, 22.3)	3512363	5670 (21.6)		
$25,000–$44,999	576 (15.7)	(14.3, 17.1)	2553714	2545 (15.9)		894 (16.3)	(15.1, 17.5)	2727946	3675 (14.5)		
$45,000–$74,999	478 (13.5)	(12.2, 14.9)	2203952	1643 (10.6)		803 (14.9)	(13.7, 16.0)	2490180	2727 (11.5)		
$75,000 and over	341 (10.0)	(8.8, 11.1)	1622926	858 (5.6)		583 (11.4)	(10.4, 12.5)	1912903	1683 (7.5)		
Do not know	129 (3.6)	(3.0, 4.2)	578665	944 (5.3)		189 (3.2)	(2.6, 3.8)	538020	1103 (4.3)		
Missing	1238 (33.8)	(31.9, 35.7)	5502547	6845 (40.4)		1835 (33.1)	(31.6, 34.6)	5531592	10324 (40.5)		
Geographic location					<0.0001					<0.0001	<0.0001
Northeast	541 (15.5)	(13.8, 17.2)	2526830	2783 (17.4)		762 (13.9)	(12.5, 15.3)	2324064	4325 (18.3)		
Midwest	850 (25.6)	(23.1, 28.1)	4161206	3642 (25.0)		1230 (26.2)	(24.2, 28.1)	4374539	5173 (23.3)		
South	1190 (32.2)	(29.7, 34.7)	5232720	6337 (37.8)		1506 (29.2)	(26.8, 31.7)	4886374	9683 (38.9)		
West	1001 (26.7)	(24.6, 28.9)	4350216	3783 (19.8)		1992 (30.7)	(28.6, 32.7)	5128027	6001 (19.6)		
Place of birth											<0.0001
US born	3164 (91.0)	(90.1, 91.9)	14807161	13116 (85.4)	<0.0001	4791 (89.8)	(88.9, 90.7)	15010846	20112 (83.5)	<0.0001	

*Note*. Column percentages shown; ^*∗*^adjusted for age, sex, and race/ethnicity based on 2000 Census data; ^*∗∗*^adjusted for age, sex, and race/ethnicity based on 2010 Census data; ^**∗∗∗**^significant changes between 2007 and 2012 indicated by nonoverlapping weighted population confidence intervals; ^W^*p* values obtained by comparing weighted estimates; note: significant changes between datasets with differing weighting schemes indicated by nonoverlapping weighted population confidence intervals as *∗∗∗*.

**Table 2:** Medical care/health characteristics of 2007 and 2012 national US samples, stratified by NVNM use in the past 12 months, National Health Interview Survey.

Characteristic	2007 (*N* = 20,127)				*p*	2012 (*N* = 30,672)				*p*	Overall *p*^W^
	NVNM use					NVNM Use					
	Yes	95% CI	Weighted population estimate^*∗*^	No		Yes	95% CI	Weighted population estimate^*∗∗*^	No		
	*N* (%)			*N* (%)		*N* (%)			*N* (%)		
Insurance status											
Uninsured	505 (13.4)	(12.4, 14.5)	2185136	2999 (16.2)	<0.0001	790 (13.0)	(12.0, 13.9)	2168607	4705 (16.5)	<0.0001	<0.0001
Medicaid	155 (4.0)	(3.3, 4.6)	645744	1510 (7.6)	<0.0001	287 (4.4)	(5.2, 10.5)	735478	2736 (9.3)	<0.0001	**<0.0001** ^**∗∗∗**^
Medicare	786 (21.6)	(20.0, 23.2)	3519561	3466 (21.7)	0.623	1277 (23.3)	(21.8, 24.8)	3892141	5922 (23.9)	0.451	<0.0001
Disability	63 (2.0)	(1.3, 2.7)	321398	165 (0.96)	<0.0001	111 (2.1)	(1.7, 2.5)	346117	258 (1.1)	<0.0001	<0.0001
Private insurance	2537 (72.6)	(70.9, 74.3)	11813273	10211 (65.3)	<0.0001	3736 (70.2)	(68.7, 71.8)	11739270	14238 (60.2)	<0.0001	<0.0001
Out of pocket costs, annual^$^					<0.0001					<0.0001	<0.0001
None	286 (7.8)	(6.8, 8.8)	1272433	2155 (11.7)		483 (8.1)	(7.3, 9.0)	1358939	3811 (13.9)		
<$500	1249 (35.0)	(33.3, 36.8)	5700791	6719 (39.6)		1780 (31.3)	(29.7, 32.9)	5230362	9593 (37.3)		**∗** **∗** **∗**
$500–$1999	1236 (34.1)	(32.4, 35.8)	5549126	4755 (29.5)		1809 (33.8)	(32.4, 35.1)	5641914	6938 (28.3)		
$2000–$2999	333 (9.3)	(8.3, 10.3)	1512573	1151 (7.7)		554 (10.3)	(9.3, 11.3)	1717635	2025 (8.7)		
$3000–$4999	217 (6.2)	(5.3, 7.1)	1008834	691 (4.5)		420 (8.3)	(7.5, 9.1)	1391128	1237 (5.1)		**∗** **∗** **∗**
$5000+	222 (6.4)	(5.6, 7.2)	1039327	635 (4.1)		396 (7.3)	(6.5, 8.1)	1219938	1180 (5.1)		
Do not know	39 (1.2)	(0.74, 1.6)	187888	439 (2.9)		48 (0.9)	(0.63, 1.2)	153088	398 (1.6)		
Delayed care because they could not afford/worried about cost	621 (17.6)	(16.2, 19.0)	2867318	1947 (12.0)	<0.0001	964 (16.7)	(15.6, 17.9)	2794500	3478 (13.2)	<0.0001	<0.0001
Perceived health status					0.001					0.001	<0.0001
Excellent/very good/good	3133 (87.7)	(86.6, 88.9)	14272846	14162 (86.3)		4849 (89.7)	(88.8, 90.6)	14987051	21383 (86.2)		
Fair	363 (10.0)	(9.0, 11.1)	1632026	1781 (10.1)		505 (8.1)	(7.3, 8.9)	1347889	2942 (10.7)		**∗** **∗** **∗**
Poor	86 (2.3)	(1.7, 2.8)	366100	602 (3.6)		136 (14.0)	(1.8, 2.7)	378064	857 (86.0)		
Physical activity^R^											
None	915 (24.6)	(22.9, 26.3)	4001744	7428 (42.2)	<0.0001	1078 (18.9)	(17.7, 20.1)	3158655	9136 (34.8)	<0.0001	**<0.0001** ^**∗∗∗**^
Low/moderate	2298 (65.3)	(63.3, 67.3)	10629710	7504 (47.5)	<0.0001	3837 (70.8)	(69.5, 72.2)	11841154	13429 (54.8)	<0.0001	**<0.0001** ^**∗∗∗**^
High	1613 (46.5)	(44.5, 48.5)	7571196	5164 (33.8)	<0.0001	2924 (54.7)	(53.0, 56.3)	9136857	9817 (40.6)	<0.0001	**<0.0001** ^**∗∗∗**^
*Mean (SE) (weekly) (min) *	*283.09 (10.23)*	*(262.96, 303.22)*		*198.45 (6.00)*	<0.0001	*325.00 (9.77)*	*(305.77, 344.24)*		*239.65 (4.63)*	<0.0001	**0.003** ^**∗∗∗**^
Body mass index (BMI)					0.016					0.031	<0.0001
<24.9	1384 (39.0)	(37.3, 40.8)	6348273	6243 (38.7)		2057 (38.6)	(37.0, 40.1)	6448785	9039 (36.3)		
25–29.9	1246 (34.5)	(32.7, 36.2)	5605830	5875 (35.4)		1916 (34.4)	(32.9, 36.0)	5757553	8717 (34.8)		
30–35	614 (17.2)	(15.8, 18.6)	2793014	2731 (16.1)		903 (16.3)	(15.1, 17.4)	2718245	4425 (17.3)		
35+	338 (9.4)	(8.3, 10.5)	1523855	1696 (9.9)		614 (10.7)	(9.8, 11.6)	1788421	3001 (11.6)		
*Mean (SE)*	*27.37 (0.11)*	*(27.15, 27.60)*		*27.35 (0.56)*	0.887	*27.54 (0.09)*	*(27.36, 27.72)*		*27.77 (0.05)*	0.024	0.2305
Tobacco use					<0.0001					<0.0001	<0.0001
Never	1894 (51.2)	(49.2, 53.2)	8330263	9977 (57.6)		3114 (56.3)	(54.8, 57.9)	9413789	14939 (58.3)		**∗** **∗** **∗**
Former	1067 (30.3)	(28.7, 31.9)	4930987	3292 (21.2)		1539 (28.6)	(27.3, 30.0)	4788193	5281 (22.0)		
Current	621 (18.5)	(16.7, 20.3)	3009722	3276 (21.2)		837 (15.0)	(13.8, 16.2)	2511022	4962 (19.6)		**∗** **∗** **∗**
Alcohol use					<0.0001					<0.0001	<0.0001
None	1006 (26.7)	(25.1, 28.4)	4349888	6958 (39.0)		1454 (24.0)	(22.5, 25.5)	4012831	9897 (36.6)		
Light	1740 (47.9)	(46.1, 49.7)	7794610	6609 (41.0)		2662 (49.6)	(48.0, 51.3)	8292892	10426 (42.7)		
Moderate to heavy	836 (25.4)	(23.6, 27.1)	4126474	2978 (19.9)		1374 (26.4)	(24.9, 27.9)	15263475	4859 (20.7)		
Substance abuse	30 (0.81)	(0.52, 1.1)	132414	96 (0.62)	0.157	62 (1.3)	(0.89, 1.6)	210988	164 (0.66)	0.0003	<0.0001
Comorbidity index^a^											
None	918 (25.6)	(23.9, 27.3)	4164042	6532 (38.4)	<0.0001	1035 (18.7)	(17.5, 19.8)	3117059	7588 (29.8)	<0.0001	**0.0001** ^**∗∗∗**^
One	912 (26.0)	(24.4, 27.5)	4225414	3846 (23.7)		1218 (22.3)	(20.9, 23.6)	3721577	5717 (22.9)		**∗** **∗** **∗**
Two	662 (18.4)	(16.9, 19.8)	2987313	2488 (15.5)		1083 (19.6)	(18.4, 20.8)	3272605	4243 (17.2)		
≥Three	1090 (30.1)	(28.4, 31.7)	4894203	3679 (22.4)		2154 (39.5)	(37.8, 41.2)	6601763	7634 (30.2)		**∗** **∗** **∗**
*Mean (SE)*	*1.83 (0.03)*	*(1.77, 1.90)*		*1.45 (0.02)*	<0.0001	*2.23 (0.03)*	*(2.17, 2.29)*		*1.84 (0.02)*	<0.0001	**<0.0001** ^**∗∗∗**^
*Chronic health conditions*											
Diabetes	314 (8.2)	(7.2, 9.1)	1331587	1633 (9.4)	0.021	596 (10.2)	(9.3, 11.2)	1709992	2965 (11.2)	0.106	**<0.0001** ^**∗∗∗**^
Kidney disease	57 (1.6)	(1.2, 2.1)	263373	289 (1.6)	0.713	91 (1.5)	(1.2, 1.8)	250872	506 (1.9)	0.047	<0.0001
GI disease^aa^	590 (16.9)	(15.5, 18.3)	2752405	1696 (10.8)	<0.0001	2186 (40.4)	(38.9, 41.9)	6744109	7468 (29.9)	<0.0001	**<0.0001** ^**∗∗∗**^
Chronic resp. conditions^b^	615 (16.9)	(15.6, 18.2)	2745606	2144 (13.4)	<0.0001	985 (18.0)	(16.8, 19.3)	3015703	3762 (15.1)	<0.0001	<0.0001
CVD^c^	257 (7.0)	(6.1, 7.9)	1142025	1078 (6.8)	0.607	393 (7.1)	(6.3, 7.9)	1186819	1829 (7.3)	0.621	<0.0001
Hypertension	1136 (31.1)	(29.4, 32.9)	5064154	4764 (28.7)	0.017	1856 (33.4)	(31.8, 35.0)	5582424	8168 (32.2)	0.165	<0.0001
Dyslipidemia	1159 (32.2)	(30.5, 34.0)	5246236	3977 (24.6)	<0.0001	1881 (33.9)	(32.2, 35.6)	5659527	6628 (26.8)	<0.0001	<0.0001
Liver disease	54 (1.5)	(1.1, 1.9)	244387	198 (1.2)	0.138	91 (1.6)	(1.2, 2.0)	271510	348 (1.3)	0.146	<0.0001
Chronic pain conditions^d^	1836 (51.6)	(49.8, 53.3)	8390744	5940 (36.9)	<0.0001	3018 (55.2)	(53.9, 56.9)	9263106	9943 (39.9)	<0.0001	**<0.0001** ^**∗∗∗**^
Mental health conditions^e^	832 (23.8)	(22.1, 25.6)	3874783	2623 (16.0)	<0.0001	1674 (30.4)	(28.9, 31.9)	5084683	5854 (22.9)	<0.0001	<0.0001
Insomnia	948 (26.9)	(25.2, 28.7)	4383811	2885 (17.8)	<0.0001	1488 (26.8)	(25.4, 28.2)	4472612	4728 (18.8)	<0.0001	<0.0001
History of cancer	355 (10.1)	(9.0, 11.2)	1638188	1101 (7.4)	<0.0001	569 (10.7)	(9.7, 11.7)	1792043	2039 (8.6)	<0.0001	<0.0001
Use of other complementary health approaches	3539 (98.9)	(98.6, 99.3)	16094998	14150 (86.2)	<0.0001	5357 (97.9)	(97.5, 98.4)	16369136	17689 (72.1)	<0.0001	**<0.0001** ^**∗∗∗**^
Other natural products^f^	3331 (93.4)	(92.4, 94.4)	15192940	10243 (64.4)	<0.0001	5215 (95.5)	(94.9, 96.2)	15965914	15806 (64.5)	<0.0001	**<0.0001** ^**∗∗∗**^
Mind-body^g^	1870 (53.2)	(51.5, 55.0)	8659604	3621 (23.6)	<0.0001	2094 (39.7)	(38.0, 41.4)	6633294	3290 (13.9)	<0.0001	**<0.0001** ^**∗∗∗**^
Manipulative/ bodywork^h^	2034 (58.4)	(56.5, 60.3)	9496114	4144 (27.1)	<0.0001	2783 (51.7)	(50.0, 53.4)	8634942	6075 (25.3)	<0.0001	**<0.0001** ^**∗∗∗**^
Energy healing/Reiki	218 (6.1)	(5.2, 7.0)	997157	137 (0.89)	<0.0001	240 (4.5)	(3.8, 5.1)	747014	123 (0.47)	<0.0001	**<0.0001** ^**∗∗∗**^
spiritual practices^i^	2334 (64.6)	(62.7, 66.5)	10512222	9637 (56.5)	<0.0001	643 (12.5)	(11.4, 13.5)	2081886	718 (2.9)	<0.0001	**<0.0001** ^**∗∗∗**^
Alternative systems^k^	858 (23.8)	(22.1, 25.5)	3867268	1136 (6.9)	<0.0001	1037 (19.0)	(17.8, 20.2)	3180077	1231 (4.9)	<0.0001	**<0.0001** ^**∗∗∗**^

*Note*. Column percentages shown; ^*∗*^adjusted for age, sex, and race/ethnicity based on 2000 Census data; ^*∗∗*^adjusted for age, sex, and race/ethnicity based on 2010 Census data; ^**∗∗∗**^significant changes between 2007 and 2012 indicated by nonoverlapping weighted population confidence intervals; ^W^*p* values obtained by comparing weighted estimates; note: significant changes between datasets with differing weighting schemes indicated by nonoverlapping weighted population confidence intervals as *∗∗∗*; ^$^family costs aside from deductibles, premiums, copays; ^R^defined as ≥10 min. increments; ^a^including diabetes, kidney disease, gastrointestinal disorder (including ulcer and/or abdominal pain, digestive allergy, and heartburn), respiratory conditions (asthma, emphysema, and/or chronic bronchitis), dyslipidemia, liver condition, rheumatoid arthritis, CVD, hypertension, nonspecific arthritis, gout, migraines, mental health condition; ^aa^including bowel conditions and ulcers (2007); past 12 months: abdominal pain, digestive allergy, heartburn, and ever having ulcer (2012); ^b^asthma, emphysema, and/or chronic bronchitis; ^c^myocardial infarction, heart disease, angina pectoris; ^d^migraines (past 3 months), nonspecific arthritis, gout, nonspecific joint pain for 3+ months, rheumatoid arthritis; ^e^depression, bipolar disorder, phobias, often anxious (past year); ^f^supplements other than NVNMs, including vitamins/minerals, probiotics (past 30 days), fish oil (past 30 days), chelation therapies; ^g^Tai Chi/Qi Gong, yoga, guided imagery, progressive relaxation, deep breathing exercises, meditation (singular term “meditation” used in 2007; expanded in 2012 to include mantra and mindfulness meditation), mindfulness-based stress reduction and cognitive therapy (2012), stress-management class (2007), biofeedback, hypnosis; ^h^practitioner-based massage (ever), chiropractic/osteopathic manipulation, Sobador visit; ^i^prayed for one's own health (2007) or centering prayer/contemplative meditation (2012); and/or visit with Espiritista (2007 only), Curandero, Shaman, and/or native American healer; ^k^acupuncture, Ayurveda, homeopathy, and naturopathy.

**Table 3:** NVNM use in two nationally representative samples of adults with diagnosed fibromyalgia, National Health Interview Survey (NHIS 2007 *N* = 221 (1.3%^*∗*C1^) versus NHIS 2012 *N* = 524 (1.7%^*∗∗*C2^)).

Characteristic	2007			2012			Overall *p*^W^
	*N* (%)	95% CI	Weighted population estimate^*∗*^	*N* (%)	95% CI	Weighted population estimate^*∗∗*^	
NVNM use ever							**<0.0001** ^**∗∗∗**^
Yes	114 (57.1)	(49.3, 64.9)	624881	204 (40.6)	(35.3, 45.8)	633029	
No	107 (42.9)	(35.1, 50.7)	469162	320 (59.4)	(54.2, 64.7)	927192	
NVNM use in past 12 months							<0.0001
Yes	82 (41.7)	(33.3, 50.2)	456477	157 (31.2)	(26.1, 36.4)	487362	
No	139 (58.3)	(49.8, 66.7)	637566	367 (68.8)	(63.6, 73.9)	1072859	
NVNM use in past 30 days							<0.0001
Yes	62 (32.7)	(24.6, 40.9)	358265	126 (24.5)	(19.9, 29.1)	382285	
No	159 (67.3)	(59.1, 75.4)	735778	398 (75.5)	(70.9, 80.1)	1177936	
Number of different NVNM supps in past 30 days^tt^							
*(Mean (SE))*	*0.99 (0.20)*	*(0.60, 1.4)*		*1.07 (0.38)*	*(0.32, 1.8)*		0.894
Physician disclosure of NVNM^d^							<0.0001
Yes	50 (64.6)	(53.1, 76.1)	294988	51 (71.4)	(59.0, 83.9)	140681	
No	32 (35.4)	(23.9, 46.9)	161489	15 (28.6)	(16.1, 41.0)	56221	

*Note*. Total sample with fibromyalgia is percentage denominator; ^*∗*^adjusted for age, sex, and race/ethnicity based on 2000 Census data; ^*∗∗*^adjusted for age, sex, and race/ethnicity based on 2010 Census data; ^*∗ ***∗∗**^significant changes between 2007 and 2012 indicated by nonoverlapping weighted population confidence intervals; ^W^*p* values obtained by comparing weighted estimates; note: significant changes between datasets with differing weighting schemes indicated by nonoverlapping weighted population confidence intervals as *∗∗∗*; ^tt^among NVNM users with fibromyalgia; including combination NVNM supplement; ^d^within past 30 days (*N* = 66; 2007); only disclosure of top therapy assessed within past 12 months (2012); ^C1^(95% CI: 1.1, 1.5); ^C2^(95% CI: 1.6, 1.9).

**Table 4:** Association of NVNM use to self-reported fibromyalgia diagnosis in two nationally representative samples of adults, National Health Interview Survey (NHIS 2007, *N* = 20,127; and 2012, *N* = 30,672).

	*N* (%)	95% CI	Unadjusted odds ratio (95% CI)	*p*	Odds ratio adjusted for demographics and related factors^*∗*^ (95% CI)	*p*	Odds ratio adjusted for demographics, lifestyle, and related factors^*¥*^ (95% CI)	*p*	Odds ratio adjusted for demographics, lifestyle, and related factors + comorbidity^t^ (95% CI)	*p*
*NHIS 2007*										
NVNM use in the last 30 days										
Fibromyalgia										
Yes	62 (3.0)	(2.1, 3.9)	3.07 (2.12, 4.45)	**<0.0001**	2.36 (1.59, 3.51)	**<0.0001**	2.54 (1.69, 3.83)	**<0.0001**	2.34 (1.55, 3.52)	**<0.0001**
No	2561 (97.0)	(96.1, 97.9)	1.00 (reference)		1.00 (reference)		1.00 (reference)		1.00 (reference)	
NVNM use in the last 12 months										
Fibromyalgia										
Yes	82 (2.8)	(2.0, 3.6)	3.09 (2.19, 4.37)	**<0.0001**	2.34 (1.58, 3.47)	**<0.0001**	2.48 (1.67, 3.71)	**<0.0001**	2.25 (1.51, 3.35)	**<0.0001**
No	3500 (97.2)	(96.4, 98.0)	1.00 (reference)		1.00 (reference)		1.00 (reference)		1.00 (reference)	
NVNM use ever										
Fibromyalgia										
Yes	114 (2.7)	(2.1, 3.3)	3.66 (2.66, 5.03)	**<0.0001**	2.85 (2.02, 4.02)	**<0.0001**	3.01 (2.13, 4.25)	**<0.0001**	2.66 (1.85, 3.82)	**<0.0001**
No	4943 (97.3)	(96.7, 97.9)	1.00 (reference)		1.00 (reference)		1.00 (reference)		1.00 (reference)	
*NHIS 2012*										
NVNM use in the last 30 days										
Fibromyalgia										
Yes	126 (3.0)	(2.4, 3.6)	2.04 (1.59, 2.61)	**<0.0001**	1.65 (1.27, 2.14)	**0.0002**	1.72 (1.33, 2.24)	**<0.0001**	1.51 (1.15, 2.00)	**0.003**
No	3994 (97.0)	(96.4, 97.6)	1.00 (reference)		1.00 (reference)		1.00 (reference)		1.00 (reference)	
NVNM use in the last 12 months										
Fibromyalgia										
Yes	157 (2.9)	(2.4, 3.4)	2.03 (1.62, 2.56)	**<0.0001**	1.72 (1.34, 2.20)	**<0.0001**	1.82 (1.42, 2.33)	**<0.0001**	1.57 (1.21, 2.04)	**0.0007**
No	5333 (97.1)	(96.6, 97.6)	1.00 (reference)		1.00 (reference)		1.00 (reference)		1.00 (reference)	
NVNM use ever										
Fibromyalgia										
Yes	204 (2.9)	(2.4, 3.3)	2.17 (1.75, 2.69)	**<0.0001**	1.84 (1.47, 2.32)	**<0.0001**	1.92 (1.52, 2.42)	**<0.0001**	1.59 (1.25, 2.03)	**0.0002**
No	7034 (97.1)	(96.7, 97.6)	1.00 (reference)		1.00 (reference)		1.00 (reference)		1.00 (reference)	

Boldface indicates statistical significance at *p* ≤ 0.05; ^*∗*^adjusted for age, sex, race, education, employment, income, geographic location of birth, insurance status (Medicaid, disability, and private), out-of-pocket medical expenses, delayed access to care due to cost, and region (2007); additionally, marital status (2012); ^*¥*^additionally adjusted for smoking status, alcohol use, exercise, and BMI (2007); and substance abuse (2012); ^t^health conditions include diabetes, kidney disease, gastrointestinal disorder (including ulcer), respiratory conditions, dyslipidemia, liver condition, rheumatoid arthritis, CVD, hypertension, arthritis, gout, migraines, and mental health condition; and self-reported health status.
